# How Is Emotional Awareness Related to Emotion Regulation Strategies and Self-Reported Negative Affect in the General Population?

**DOI:** 10.1371/journal.pone.0091846

**Published:** 2014-03-17

**Authors:** Claudia Subic-Wrana, Manfred E. Beutel, Elmar Brähler, Yve Stöbel-Richter, Achim Knebel, Richard D. Lane, Jörg Wiltink

**Affiliations:** 1 Department of Psychosomatic Medicine and Psychotherapy, Universal Medical Center Mainz, Mainz, Rhinland-Palatinate, Germany; 2 Department of Medical Psychology and Medical Sociology, University Leipzig, Leipzig, Saxony, Germany; 3 Department of Neuroscience, College of Social and Behavioral Sciences, The University of Arizona, Tucson, Arizona, United States of America; University of Adelaide, Australia

## Abstract

**Objective:**

The Levels of Emotional Awareness Scale (LEAS) as a performance task discriminates between implicit or subconscious and explicit or conscious levels of emotional awareness. An impaired awareness of one's feeling states may influence emotion regulation strategies and self-reports of negative emotions. To determine this influence, we applied the LEAS and self-report measures for emotion regulation strategies and negative affect in a representative sample of the German general population.

**Sample and Methods:**

A short version of the LEAS, the Hospital Anxiety and Depression Scale (HADS) and the Emotion Regulation Questionnaire (ERQ), assessing reappraisal and suppression as emotion regulation strategies, were presented to N = 2524 participants of a representative German community study. The questionnaire data were analyzed with regard to the level of emotional awareness.

**Results:**

LEAS scores were independent from depression, but related to self-reported anxiety. Although of small or medium effect size, different correlational patters between emotion regulation strategies and negative affectivity were related to implict and explict levels of emotional awareness. In participants with implicit emotional awareness, suppression was related to higher anxiety and depression, whereas in participants with explicit emotional awareness, in addition to a positive relationship of suppression and depression, we found a negative relationship of reappraisal to depression. These findings were independent of age. In women high use of suppression and little use of reappraisal were more strongly related to negative affect than in men.

**Discussion:**

Our first findings suggest that conscious awareness of emotions may be a precondition for the use of reappraisal as an adaptive emotion regulation strategy. They encourage further research in the relation between subconsious and conscious emotional awareness and the prefarance of adaptive or maladaptive emotion regulation strategies The correlational trends found in a representative sample of the general population may become more pronounced in clinical samples.

## Introduction

An association between maladaptive emotion regulation strategies and elevated negative affect was shown by experimental studies exploring the impact of diverse emotion regulation strategies on negative affect and by surveys in clinical groups asking for the relation of self-reported emotion regulation strategies to affective state. However, these studies neither examined if their participants were able to experience affective arousal consciously as feeling states, nor did they assess if impairments in the conscious awareness of emotions were related to specific emotion regulation strategies which may or may not increase the risk of elevated negative affect and its negative effects on mental and physical well-being. The Levels of Emotional Awareness Scale [1], a performance task based on the Levels of Emotional Awareness (LEA) Theory by Lane & Schwartz [2] differentiates between implicit or preconscious levels of emotional awareness, e.g. affective arousal expressed as bodily sensation or action tendency, and explicit emotional awareness, e.g. the ability to be consciously aware of one's emotions and to express them verbally as feeling states. In order to explore if the level of emotional awareness has an impact on the preferred emotion regulation strategy and the related intensity of negative affect, we administered a short version of the LEAS and self-report questionnaires on emotion regulation strategies and negative affect (depression and anxiety) to a large representative sample of the German general population.

The authors are aware that negative affectivity as a broader concept differs from anxiety and depression as specific and distinct sets of negative cognitions and emotions. To make the paper reader-friendly, we subsummize the affective components of depression and anxiety and the ability to put these affective components into verbal expressions of variable differentiation under the terms “negative affect” or “negative affectivity”

There is a large body of evidence demonstrating that adaptive emotion regulation strategies like reappraisal, problem solving or acceptance of emotions are related to lower levels of negative affectivity, especially to less anxiety and depression. This is true for experimental settings with healthy subjects, where instructions to regulate anxious arousal - evoked by a performance task (e.g. giving a speech in front of a video camera) - by reappraisal or acceptance reduce physiological arousal, improve memory of the performed speech and decrease negative affect compared to instructions to suppress the anxious arousal [3], [4]. Subjects suffering from anxiety and mood disorders also reported less negative affect after an experimental induction of negative affectivity (watching a sad film) when instructed to use reappraisal as an emotion regulation strategy [5]. In studies on persons confronted with real threats, e.g. in severe medical conditions like HIV-infection or need for haemodialysis, reappraisal in particular was related to lower levels of anxiety or depression [6], [7]. In persons confronted with infertility or the hardships of aging, acceptance or reappraisal of the condition led to a decrease in negative affectivity [6], [8], while a lack of reappraisal was related to increased emotional eating in women with eating disorders [9].

On the other hand, an association between suppression as a maladaptive emotion regulation strategy and heightened negative affectivity has been observed in numerous studies. In experimental settings inducing negative affect, suppression as an emotion regulation strategy led to higher negative affectivity in participants recovered from depression or suffering from anxiety and mood disorders [5], [10]. While suppression leads to a short-term reduction of sadness in persons suffering from depression [11], it seems to foster the development of depressive symptoms “in the long run” [12], [13]; suppression also predicted PTSD-symptoms in a trauma-exposed sample [14] and was related to higher stress-related symptomatology [15]. A recent meta-analysis of 114 studies on the relation between emotion regulation and psychopathology found a negative association with reappraisal, problem solving and acceptance of emotions and a positive association between negative affectivity and suppression, avoidance and rumination. [16].

Especially when studying the relation between emotion regulation strategies, negative affectivity and psychopathological states, the association between emotion regulation and emotional awareness becomes a matter of interest. Anxiety as well as depression consist of typical patterns of physiological arousal, action tendencies (e.g. fight or flight in anxious arousal) and consciously experienced feeling states (e.g. sadness in depression). Conscious awareness of the components that constitute a state of anxiety or depression may differ between individuals, contributing to individual differences in the experience of these mood states. In one person an anxious arousal may lead to a state of restlessness, but the conscious experience of anxiety may be lacking, while another person is able to connect bodily signs of anxious arousal (e.g. nausea) to consciously experienced anxiety. In depressed mood one person may avoid social contacts, but may state that he or she does not feel anything but exhaustion, while another person may be able to connect his or her urge for a social retreat with feelings of sadness and guilt. For the employment of emotion regulation strategies, the composition of these individual blends of the awareness of negative affective arousal may be crucial. Reappraisal as an adaptive emotion regulation strategy requires thinking about how one is experiencing a situation in order to develop a new approach to it. This will include the necessity to become consciously aware of one's emotions. Suppression as a maladaptive emotion regulation strategy is a conscious decision not to explore one's reaction to a certain situation in depth, therefore it does not necessarily require the conscious awareness of one's feelings. Experiencing a diffuse negative arousal may be sufficient to employ suppression in order to reduce negative tension (although another individual may also suppress contemplating a consciously experienced feeling like sadness). Thus, in order to explore the relation between emotion regulation strategies and negative affectivity, it may be crucial to evaluate the capacity of an individual for emotional awareness. In this regard, it may be of special interest to differentiate whether a person habitually processes affective arousal on a more implicit level, e.g. via action tendencies or by being aware of global states of positive or negative tension, or on an explicit level that includes the conscious awareness of distinct emotions that can be put into words.

The Levels of Emotional Awareness (LEA) theory [2] provides a theoretical background for the differentiation between implicit and explicit levels of emotional awareness. The LEA-theory holds that the ability to be consciously aware of one's feelings results from a process of cognitive-emotional development, which is parallel to the stages of sensory-cognitive development as described by Piaget [17]. In normative development, a constant process of differentiation and generalization leads from the implicit, preconscious expression of affective arousal as bodily sensations (LEA level 1) and action tendencies or global states of positive or negative tension (LEA level 2) to the explicit representation of affective arousal as distinct, consciously experienced feeling states (conscious awareness of one feeling at one time: LEA level 3; a blend of feelings at one time: LEA level 4; awareness of different blends of feelings in self and other: LEA level 5). From this viewpoint, individuals may differ in the level of emotional awareness they are capable of; disruptions in the somato-psychic development may lead to impairments in emotional awareness. The Levels of Emotional Awareness Scale (LEAS) [1], a performance measure with proven reliability and construct-validity [18], has been constructed on the theoretical background provided by the LEA-theory and assesses these levels of emotional awareness. The LEAS has been applied to the field of psychosomatic and psychotherapy research; compared to healthy individuals, patients with somatoform disorders, eating disorders, depressive states, alcohol addiction and functional psychosomatic conditions, e.g. essential hypertension, demonstrated lower levels of emotional awareness [19], [20], [21], [22], [23], [24]. Lower levels of emotional awareness have been linked to impairments in mentalization, which is a more general capacity to represent psychic contents consciously and explicitly as thoughts, feelings and intentions [25], and there is evidence that multimodal inpatient psychotherapy has the capacity to transform emotional awareness from an implicit to an explicit level [25], [26].

Based on the levels of emotional awareness theory the LEAS offers an opportunity to study the impact of the level of psychic representation of affective arousal on the preferred emotion regulation strategy. The assessment of the level of emotional awareness may help us to understand the preconditions that lead an individual to favor adaptive or maladaptive emotion regulation strategies and therefore heighten or lower the risk of negative affectivity. In order to explore if the level of emotional awareness mediates the association between different types of emotion regulation strategies and self-assessed depression and anxiety as psychic symptoms that are closely related to negative affectivity, we applied a short version of the LEAS, a questionnaire asking for reappraisal and suppression as emotion regulation strategies (ERQ) and a measure for the self-assessment of anxiety and depression (HADS) to a large, representative sample of the German general population (n = 1844 complete data sets). In concordance with the existing body of evidence, we expected: a) a positive relationship between suppression as a maladaptive emotion regulation strategy and elevated negative affectivity, b) a negative relationship between reappraisal as an adaptive emotion regulation strategy and negative affectivity.

## Sample and Methods

### Sample

A nationwide survey representative of the German general population was conducted by employing an institute certified for demographic research (USUMA GmbH  =  Unabhängige Serviceeinrichtung für Umfragen, Methoden und Analysen; independent service for surveys, methods and analyises in market and social research, limited liability commpany) according to the German law of data protection (§30a BDSG) and with written consent. Previously ethics were weighted to the respective interests of the public and of the individuals concerned following §823 (BGB) of the Civil Code of Law and in accordance with the guidelines in the Declaration of Helsinki. Age, gender, and educational level were the major criteria for representativeness; the age and gender distribution of our data set is comparable to the data of the German Federal Bureau of statistics.

The set of questionnaires was administered to the final sample of 2524 persons. In the first wave, attempts were made to contact 4630 persons. Two callbacks had to be without success before an address was considered a failure. From the 4630 selected addresses, 4572 were valid. The three-stage sampling procedure consisted of 258 sample points in the first, household in the second, and persons in the third stage. Target households within the sample points were determined using the random-route procedure; the target persons within the households were selected using random digits. The basic population for the data collection is made up of the German population aged at least 14 years and living in private households. The data sets for individuals aged at least 18 years were used for the data evaluation. The survey was conducted in May/June of the year 2009.

**Figure 1:**
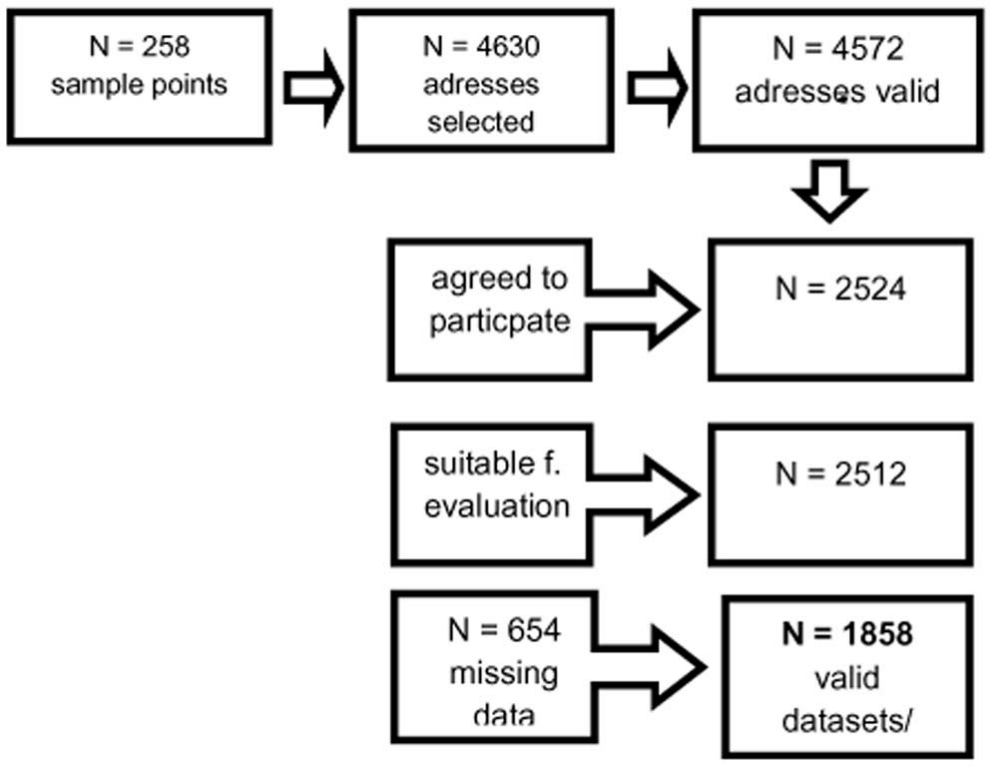
Flow chart illustrating the data collection.

The representativeness of the sample was ensured by drawings of ADM (Arbeitskreis Deutscher Marktforscher: Working party of demoscopic market research) samples und by comparison with the data of German Federal Statistical Office. All participants were visited by an interviewer who informed about the investigation and provided written informed consent. The interviewer waited until participants answered all questionnaires and offered help in case of difficulties to understand single questions. If not at home, up to three attempts were made to contact the selected person. A total of 2524 persons agreed to participate (55.2% of the valid addresses) and 2512 interviews and questionnaires were suitable for evaluation. Because of missing data the responses of 654 (14.1% from the 4572 valid addresses) participants could not be analyzed. Main reasons were incomplete answers to either the self-assessment questionnaires ERQ or HADS or the performance measure LEAS, given in a 4 item short version. A total of 1858 participants were included into further analysis (see flow-chart, figure 1).

The mean age of the participants was 48.3 (SD 18.0) years. 56.1% were female. The age-groups were represented in comparable proportions: 14 to 24 years: 10.4%, 25 to 34 years: 15.4%, 35 to 44 years: 18.1%, 45 to 54 years: 18.4%, 55 to 64 years: 15.8%, 65 to 74 years: 14.0% and 75 years and older: 8.0%. The age and gender distribution is comparable to the data of the German Federal Bureau of statistics.

Approximately one half (50.8%) of the participants were married; 56.1% lived in a partnership. The great majority (82.7%) had a low or average educational level. About 44.2% had a household income above 2000 Euro per month (December 2013: relation Euro to US-$ 1 to 1,38). Over one third (39.6%) of the participants worked fulltime, 27.2% were retired and 7.8% unemployed. 21.5% lived in the eastern federal states of Germany.

### Assessment of the Level of Emotional Awareness

In 1990 Lane and colleagues presented the first normative data of the Levels of Emotional Awareness Scale (LEAS), a paper and pencil performance measure developed according to the Levels of Emotional Awareness (LEA)-theory [2]. The LEAS consists of 20 emotion-provoking vignettes. Subjects are asked to write down in a free text how he/she would feel in the place of the protagonist of the vignette and how the other person involved in the described scene would feel. The scoring rules are established in regard to the LEA-theory; scores are given for “self” (subject in the place of the protagonist) and “other” (subjects description how the other person involved in the scene would feel) from which a total score is derived. Scoring is assisted by a glossary which links affect-related words to the appropriate LEA-levels. A LEAS-score of 1 indicates that affective arousal is described by bodily sensations or that the subject stated that he/she does not know how he/she would feel; a score of two puts affective arousal in terms of an action tendency or global expressions of negative or positive arousal (e.g. “good” vs. “bad”); a score of 3 indicates that affective arousal is expressed by an emotion word; a score of 4 is given when a mixture of feelings is described and a score of 5 is given when for “self” and “other” different states of mixed feelings are described. Levels 1 and 2 represent implicit states of emotional awareness, levels 3 to 5 represent explicit states of emotional awareness which enable the subject to be consciously aware of feeling states and to express them verbally. The distinction between implicit and explicit levels of emotional awareness is theoretically derived by paralleling the cognitive-emotional development (LEA-theory) to the cognitive-sensory development as described by Piaget [17]. Explicit levels of emotional awareness are therefore comparable to the state of concrete-operational intelligence, the ability to express emotions symbolically by emotion words is comparable to the ability to be aware of the invariance of objects even when they change some of their characteristics and to categorize them as similar (e.g. dogs and cats are both animals). This theoretically derived distinction has been supported empirically by demonstrating that patients with somatoform disorders who are characterized by a diminished ability to be consciously aware of their feelings scored – as hypothesized – in the implicit levels of emotional awareness while patients with other diagnoses (e.g. eating disorders or depression) or healthy controls scored significantly higher [25], [26]. An example of a LEAS-vignette and answers at the 5 different LEA-levels is presented in table 1. The 20-items LEAS has a maximum score of 100 with scores between 0 and 20 indicating LEAS level 1 and scores between 81 and 100 indicating LEAS level 5. The 20-items LEAS can be divided into two half-forms with 10 items each which are statistically paralleled. The normative data on the LEAS collected in an US community sample demonstrated good psychometric properties of the scale, the correlation with measures applied to test its construct validity was sufficient [1], [18]. To date LEAS-versions are available in French, German and Japanese. In all languages studies with clinical and populations samples have been conducted and revealed similar correlational patterns with other psychological measures or sociodemographic data as already described by Lane and colleagues in 1990 and 1996; for an overview see[27]. Table 1 gives an example of an item of the LEAS and its scoring.

**Table 1:** LEAS Item 20, selected answers and scores (example according to Lane, 1990).

LEAS Item 20:		
*“You and your best friend are in the same line of work. There is a prize given annually to the best performance of the year. The two of you work hard to win the prize. One night the winner is announced: your friend. It is your friend. How would you feel? How would your friend feel?”*		
Level 1 = bodily sensation	*I would feel ill*	Score: 1
Level 2 = action tendency and undifferentiated affect state	*I would like to run away. My friend would feel good.*	Score: 2
Level 3 = singel emotion	*We both would be happy*	Score: 3
Level 4 = blend of emotions	*I would feel depressed, but would be happy that my friend won the prize. My friend would be happy.*	Score: 4
Level 5 = blend of blends of emotions	*I would feel disappointed. But if someone else won, I would be happy that it is my friend. My friend will be proud and happy, but also concerned about me.*	Score: 5

To apply and score one of the 10-items version of the LEAS to a sample with over 2000 participants would be overly time consuming and costly; therefore we constructed a 4-items version of the LEAS for the purposes of our study.

### Construction and preliminary evaluations of the 4 item LEAS

#### Step 1

In a *first step* we referred to the normative US community sample of the LEAS [1], [18] to identify items suited for the 4-items LEAS. The US community sample consisted of 380 participants (184 male, 196 female) distributed equally over the age groups. Exclusion criteria for participation were severe mental disorders, substance abuse and cognitive impairment. The LEAS was completed in presence of an interviewer available for questions. Participants were paid $10 for compensation. Previously these data have been used in collaboration with Richard Lane to establish that the two half forms of the 20 items LEAS are statically paralleled [26]. Now, we conducted item analyses for both halves separately. The four items with the highest discriminatory power of each version were chosen for further evaluation. Internal consistencies (Cronbach's alpha) for the LEAS 4 item version A was alpha = 0.684, for LEAS 4 item version B alpha = 0.683; the two extracted versions correlated with r = 0.660. Correlations between the LEAS 4-item versions and their corresponding LEAS 10 items version were r = 0.891 for A and r = 0.875 for B. Both 4 item scales also correlated strongly with the original 20 item version: A: r = 0.853, B: r = 0.844.

#### Step 2

In a *second step* we evaluated the 4 Items versions in the dataset of the validation study of the German 10 item versions of the LEAS [28]. The sample consisted of 331 students with a mean age of 23 years. 51.7 percent were female. Correlations between the 4 items LEASs and their corresponding 10 item LEAS versions were high: A r = 0.895, B r = 0.879.

#### Step 3

Psychometric properties of the two 4 item versions were tested in a new sample of students (N = 126; mean age 26 years; 62% female) who were only presented with the 4 items LEAS. 54 students completed Version A, and 72 completed version B. Participants were paid €5 for compensation. The mean scores of both 4 items LEAS versions did not differ significantly (T(124) = 0.077, n.s.). Nevertheless, in Version B we could not find relationships to other measures as reported previously [8]. Against our expectations, the 4 item LEAS B correlated with the TAS-20 [29], [30], [31], an established self-report measure for alexithymia (N = 71, r = −0.350, p = 0.003) and with the STAI, a self-report questionnaire for state anxiety [32], [33] (N = 72, r = 0.250, p = 0.034). As expected, both versions were not related to distress (GSI, SCL90-R) [34]; German version [35]. Because of the unexpected correlations with anxiety and the TAS-20, we rejected Version B and pursued further evaluation of version A.

#### Step 4

To test the properties of the 4-item LEAS in a clinical sample, we assessed 80 psychosomatic inpatients with the scale at intake. The mean age of the patient sample was 43 years (66% female). We found no significant correlations between the LEAS, the global score of the SCL 90-R, and TAS-20. The TAS-20 was moderately related with the STAI (N = 79, r = 0.252, p = 0.025), and strong with the global score of the SCL 90-R (N = 78, r = 0.578, p<.001)

### Hospital Anxiety and Depression Scale (HADS)

The *Hospital Anxiety and Depression Scale (HADS)* was administered to assess anxiety and depression. The two factor analytically derived scales of the HADS are represented by seven items each (example for an anxiety item: “I get sudden feelings of panic”). The answers range from 1 “very often indeed” to 4 “not at all”. Good internal consistencies for both scales could be demonstrated for the German version of the HADS (Cronbach's alpha: anxiety 0.80; depression 0.81) [36], [37], [38].

### Emotion Regulation Questionnaire (ERQ)

The German Version of the Emotion Regulation Questionnaire (ERQ) [39] has recently been published [40]. The ERQ assesses two common emotion regulation strategies with a 10 item self-report instrument: expressive suppression and reappraisal of aversive emotions. Internal consistencies of both scales were in the range of the original version (Cronbach's alpha = 0.74 for suppression and alpha = 0.76 for reappraisal). We applied the modified scoring algorithm, as evaluated by Wiltink et al. [41], allowing a cross-loading of item 8. Particularly, the two factor structure (reappraisal and suppression) of the American original could be replicated. Abler and Kessler [40] found a positive relation between suppression and the depression scale of the SCL 90-R.

### Statistical analyses

For parametric analyses we used t-tests, Pearson correlations, and partial correlations (controlling for age). In order to compare correlation coefficients we performed the Fisher r-to-z transformation. To further test our finding that the LEAS mediates the association between emotions and emotion regulation, we conducted an additional path-analysis. We chose a multiple-group model, differentiating between participants with implicit and explicit emotion representations, based on their LEAS-scores (low vs. high). Because of the cross-sectional nature of our data, we refrained from causal hypotheses regarding the interrelation of the HADS and the ERQ and postulated only correlational associations. We included several predictor variables: age, level of education, total household income, and living in a partnership. The analysis started with a saturated model with zero degrees of freedom and all parameters set to be equal between both groups. Insignificant pathways were deleted as long as the decrease in the overall model fit was insignificant. The allowance for the groups to differ from each other was only allowed if the overall fit significantly increased thereby (based on Chi^2^-difference tests between the nested models). With regard to the allowance of group differences, the same procedure was applied to the mean structure of the model. The resulting structural relations are depicted in Figure 1. We report the overall Chi^2^ test of the model (where insignificance is indicative of a good fit to the data) and the RMSEA (where values below .08 indicate an acceptable fit and values below .05 a good fit to the data). The overall Chi^2^ tends to be too conservative (especially in large samples even the smallest differences can lead to significant results), an alternative procedure is based on the ratio of the Chi^2^ to the remaining degrees of freedom (the fit is considered to be adequate if this ratio is ≤2.5). The statistical computations were performed with SPSS (Version 17.0) and the path-analysis with LISREL (8.72).

The level of significance was set to p<.05. We did not adjust alpha because of the exploratory nature of our analyses regarding the relationship between the LEAS, explicit emotion regulation, and negative affectivity.

## Results

### Reliability

We determined *inter-rater reliability* by Intra-Class-Correlations (ICC 3.1, two-way mixed, single measure) for a total of 48 subject who completed the LEAS from the German representative sample. These LEAS were rated independently by two trained raters (C. Subic-Wrana and D. Böhringer). ICCs between the two raters for the single items ranged from 0.690 und 0.915. For the total scale (4 items), a very good agreement of ICC = 0.898 was achieved. The Pearson correlation between raters was r = 0.917. *Internal consistency* (Cronbach's alpha) was alpha = 0.615 for the 4 item short form of the LEAS. A principal components analysis was performed to investigate the factor structure of the 4-item LEAS. Only one factor with an eigenvalue greater than 1.0 was identified. The factor explained 47% of the total variance. All 4 items had high factor loadings (between 0.68 and 0.69) on this general factor. Thus, we assume that all 4 items measure the same concept, which is also reflected by the comparably high internal consistency of the instrument.

### Level of Emotional Awareness

With a total score of 9.92 (sd 3.14) and a mean score of 2.48 (range: 0 to 4.75) the German representative sample demonstrates an overall emotional awareness between the implicit (level 1 to 2) and explicit (level 3 to 5) levels. The majority of the sample (N = 1325) achieved mean scores that indicate explicit levels of emotional awareness, i.e. the ability to experience emotions consciously and to express them verbally (total score >/ = 16, mean >/ = 4); N = 533 participants had mean scores that indicate implicit levels of emotional awareness (total score </ = 12; mean </ = 3). Women (total score: 10.23; sd 3.02; mean 2.56 with a range from 0 to 4.75) scored higher than men (total score: 9.53; sd 3.24; mean 2.38 with a range from 0 to 4.75), and this difference was significant (T(1687.3) = 4.732, p<.001). More detailed information is provided in tables 2 to 7.

**Table 2:** Correlations between LEAS, anxiety, depression, emotion regulation and age: total (N = 1858), male (N = 815); female (N = 1043).

	LEAS		HADS-D		HADS-A		ERQ-R		ERQ-S		Age		M (SD)
LEAS	**f**	**m**	*−0.036*		0.102**		0.062*		−0.137**		−0.089**		*9.92 (3.14)*
HADS-D	−0.019	−0.050	**f**	**m**	0.673**		−0.152**		0.188**		0.259**		*4.55 (3.91)*
HADS-A	0.112**	0.069*	0.698**	0.652**	**f**	**m**	−0.090**		0.105**		0.085**		*4.68 (3.66)*
ERQ-R	0.059	0.057	−0.150**	−0.153**	−0.116**	−0.064	**f**	**m**	0.179**		*−0.028*		*24.47 (5.90)*
ERQ-S	−0.167**	−0.088*	0.178**	0.200**	0.121**	0.095**	0.144**	0.230**	**f**	**m**	0.079**		*17.72 (5.10)*
**Age**	0.116**	−0.060	0.262**	0.255**	0.058	0.120**	−0.012	−0.048	0.073*	0.088*	f	m	**48.28 (17.96)**
**M (SD)**	**10.23 (3.02)**	9.53 (3.24)	4.48 (3.87)	4.65 (3.97)	4.97 (3.73)	4.30 (3.53)	24.65 (5.86)	24.23 (5.94)	17.47 (5.16)	18.04 (5.02)	48.43 (18.06)	48.10 (17.84)	

Pearson correlations; ** p<.001;* p<.05

Top part  =  all participants  =  numbers in italics, bottom part  =  separated by gender: male  =  columns marked **m**; female  =  columns marked **f**

LEAS = Levels of Emotional Awareness Scale; HADS-D  =  Hospital Anxiety and Depression Scale (Depression), HADS-D  =  Hospital Anxiety and Depression Scale (Anxiety), ERQ-R  =  Emotion Regulation Questionnaire (Reappraisal), ERQ-S  =  Emotion Regulation Questionnaire (Suppression)

**Table 3:** Correlations between negative affect (anxiety, depression) and emotion regulation depending on level of LEAS: level 1+2 (N = 533).

	HADS-D	HADS-A	ERQ-R	ERQ-S	M (SD)
**LEAS**	0.031	0.021	−0.008	−0.055	6.00 (2.09)
**HADS-D**		0.694**	−0.091*	0.209**	4.68 (4.07)
**HADS-A**			0.000	0.157**	4.18 (3.56)
**ERQ-R**				0.208**	23.88 (5.49)
**ERQ-S**					18.28 (4.64)

Partial correlations (controlled for age); ** p<.001;* p<.05; LEAS level 1+2 (age: M = 50.01; 17.73).

**Table 4:** Correlations between negative affect (anxiety, depression) and emotion regulation depending on level of LEAS: level 3–5 (N = 1325).

	LEAS	HADS-D	HADS-A	ERQ-R	ERQ-S
**HADS-D**	−0.038				
**HADS-A**	0.080	0.675**			
**ERQ-R**	0.028	−0.174**	−0.128**		
**ERQ-S**	−0.159**	0.162**	0.088**	0.180**	
**M (SD)**	11.50 (1.85)	4.50 (3.85)	4.88 (3.68)	24.70 (6.04)	17.49 (5.27)

Partial correlations (controlled for age); ** p<.001;* p<.05; LEAS level 3–5 (age: M = 47.59; 18.01)

**Table 5:** Correlations between negative affect (anxiety, depression) and emotion regulation for all participants.

A) Total (N = 1858)						
	LEAS	HADS-D	HADS-A	ERQ-R	ERQ-S	M (SD)
**LEAS**		−0.013	0.110**	0.059*	−0.130**	9.92 (3.14)
**HADS-D**			0.676**	−0.150**	0.174**	4.55 (3.91)
**HADS**-**A**				−0.088**	0.099**	4.67 (3.66)
**ERQ-R**					0.182**	24.47 (5.90)
**ERQ-S)**						17.72 (5.10)

Partial correlations (controlled for age); ** p<.001;* p<.05

LEAS = Levels of Emotional Awareness Scale; HADS-D  =  Hospital Anxiety and Depression Scale (Depression), HADS-D  =  Hospital Anxiety and Depression Scale (Anxiety), ERQ-R  =  Emotion Regulation Questionnaire (Reappraisal), ERQ-S  =  Emotion Regulation Questionnaire (Suppression)

**Table 6:** Correlations between negative affect (anxiety, depression) and emotion regulation depending on gender and level of LEAS (1+2).

Level 1+2: male (N = 268, in italics), female (N = 265, underlined)						
	LEAS	HADS-D	HADS-A	ERQ-R	ERQ-S	M (SD)
**HADS-D**	0.001		0.674**	−0.173*	0.163*	*5.82 (2.18)*
**HADS-A**	0.012	0.726**		*−0.049*	*0.051*	*4.97 (4.21*
**ERQ-R**	−0.068	−0.007	0.047		*0.093*	*4.10 (3.58)*
**ERQ-S**	−0.067	0.260**	0.266**	0.318**		*23.92 (5.33)*
**M (SD)**	6.19 (1.99)	4.38 (3.91)	4.27 (3.54)	23.83 (5.66)	18.21 (4.63)	*18.37 (4.65)*

Partial correlations (controlled for age); ** p<.001;* p<.05; male  =  in italics; female  =  underlined.

LEAS = Levels of Emotional Awareness Scale; HADS-D  =  Hospital Anxiety and Depression Scale (Depression), HADS-D  =  Hospital Anxiety and Depression Scale (Anxiety), ERQ-R  =  Emotion Regulation Questionnaire (Reappraisal), ERQ-S  =  Emotion Regulation Questionnaire (Suppression)

**Table 7:** Correlations between negative affect (anxiety, depression) and emotion regulation depending on gender and level of LEAS (3–5).

Level 3-5: male (N = 547, in italics), female (N = 778, underlined)						
	LEAS	HADS-D	HADS-A	ERQ-R	ERQ-S	M (SD)
**LEAS**		*−0.045*	0.091*	*0.041*	−0.110*	*11.35 (1.81)*
**HADS-D**	0.035		0.639**	−0.133*	0.193**	*4.49 (3.83)*
**HADS-A**	0.060	0.705**		*−0.065*	0.104*	*4.40 (3.51)*
**ERQ-R**	0.015	−0.205**	−0.179**		0.292**	*24.39 (6.22)*
**ERQ-S**	−0.186**	0.141**	0.090*	0.105*		*17.89 (5.19)*
**M (SD)**	11.60 (1.86)	4.51 (3.86)	5.21 (3.77)	24.92 (5.91)	17.22 (5.30)	

Partial correlations (controlled for age); ** p<.001;* p<.05; male  =  in italics; female  =  underlined. LEAS = Levels of Emotional Awareness Scale; HADS-D  =  Hospital Anxiety and Depression Scale (Depression), HADS-D  =  Hospital Anxiety and Depression Scale (Anxiety), ERQ-R  =  Emotion Regulation Questionnaire (Reappraisal), ERQ-S  =  Emotion Regulation Questionnaire (Suppression).

### Relationships between emotional awareness (LEAS), distress (HADS, anxiety and depression), and self-assessed emotion regulation strategies (ERQ)

In the total sample, low but significant correlations indicated trends that related the level of emotional awareness positively with reappraisal and self-reported anxiety and negatively with age and supression; there was no significant correlation between the level of emotional awareness and depression. Depression and anxiety were associated positively with suppression and negatively with reappraisal, (compare Table 2).

### Implicit emotional awareness (LEAS Levels 1 and 2 =  total score </ = 12): Relationships between negative affect (HADS, anxiety, depression) and emotion regulation (ERQ)

In order to analyse the associations between low emotional awareness, negative affect and self-reported emotion regulation strategies, we performed partial correlations to control for the relation between LEAS and age that had been revealed in the statistical analyses for the group as a whole; differences regarding sex were explored by performing separate statistical analyses for men and women. In the participants with implicit emotional awareness (N = 533), suppression and negative affect (depression r = 0.209, p<.001; anxiety r = 0.157, p<.001) correlated positively; for reappraisal there was only a small, but significant, negative correlation with anxiety (r = −0.091, p<.05) (table 3 and 4). In women there was a more pronounced relation between suppression and both negative affect states (comparison of the correlation between depression and suppression in women with implict emotional awareness r = 0.260, p<.001 and explizit emotional awareness r = 0.141, p<.001 by Fisher r-to-z transformation: z = 1.75, p = .082; comparison of the correlation between anxiety and suppression in women with implicit emotional awareness r = 0.266, p<.001 and with explict emotional awareness r = 0.090, p<.05 by Fisher r-to-z transformation: z = 2.55, p = .011); in men depression correlated low and positively with suppression (r = 0.163, p<.05) and low and negatively with reappraisal (r = −0.173, p<.05) (table 5 to 7).

### Explicit emotional awareness (LEAS Levels 3 to 5 =  total score </ = 16): Relationships between negative affect (HADS, anxiety, depression) and emotion regulation (ERQ)

Because of the association between LEAS and age, we controlled all analyses for age including explicit levels of emotional awareness (N = 1325). In this group negative affect correlated negatively with reappraisal (depression r = −0.174, p<.001, anxiety r = −0.128, p<.001) and positively with suppression (depression r = 0.162, p<.001, anxiety r = 0.088, p<.001); there was also a negative correlation between LEAS-scores and suppression (r = −0.159, p<.001); all reported correlations were low (table 3 and 4). However, Fisher r-to-z transformations revealed the negative correlation between depression and reappraisal in women with explicit emotional awareness as significantly stronger as in women with implict emotional awareness (z = 2.81, p = .005), for the negative correlations between reappraisal and anxiety (z = −1.87, p = .062) and between LEAS and suppression (z = 1.69, p = .091) in women there was a trend in favor of the explict awareness-group (women/reappraisal: depression r = −0.205, p<.001, anxiety r = −0.170, p<.001; LEAS-suppression: women r = −0.186, p<.001).

### Correlation of the subscales of HADS and ERQ

The subscales of the ERQ and the HADS were moderately to highly interrelated; all findings are reported in tables 2 to 7.

### Path-Analysis

**Figure 2:**
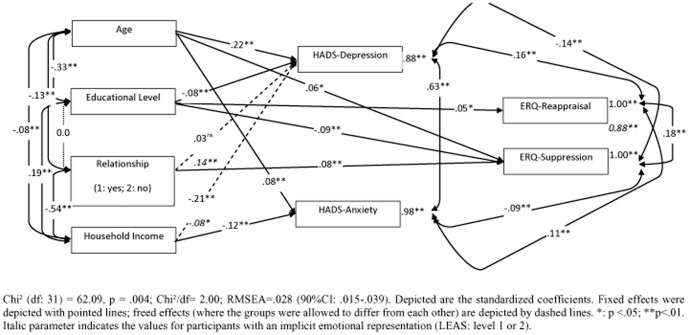
Influences of implicit vs. explicit emotional awareness on sociodemographic variables, negative affect, and emotion regulation.

In a path-analysis we explored the relation between sociodemographic variables, negative affect and emotion regulation strategies; a simultaneously applied multi-group model explored if these relations differed between subjects with implicit and explicit emotional awareness (Figure 2). Overall, the effects were in the low to medium range. In the multiple group model, subjects with implicit emotional awareness had less variability in both emotion regulation strategies than subjects with explict emotional awareness. Living without partnership was only related to an increase of depression in subjects with implicit emotional awareness (implicit emotional awareness: β = .14, p<.01; explicit emotional awareness: β = .03, ns). Higher depression scores were also related to lower household income, this association was stronger in subjects with explicit emotional awareness (β = −.21, p<.001; implicit emotional awareness: β = −.08, p<.05).

## Discussion

To our knowledge, this is the first study exploring the association between the Level of Emotional Awareness Scale as an established measure of implicit and explicit representations of affective arousal, self-described and therefore consciously available emotion regulation strategies, and self-reported negative affect in a large representative sample of the general population in a Western country.

With a total score of 9.92 (sd 3.14) and a mean of 2.48 our representative German community sample scores between implicit or subconscious (level 1 and 2) and explicit or conscious (levels 3–5) emotional awareness. The sample demonstrated relationships between the LEAS and sociodemographic variables comparable to the literature: women scored higher than men [28], [42] and age was negatively correlated with LEAS scores [18], [42]. We found a positive relation between suppression as a maladaptive emotion regulation strategy and self-reported negative affect, higher age seems to foster this relation. Reappraisal as an emotion regulation strategy was related to less self-reported negative affect, participants with higher levels of emotional awareness reporting less negative affectivity. These findings are in line with numerous findings in clinical and non-clinical samples, e.g. the meta-analysis of Aldao and collegues [16], it has to be noted that the relations we found were only of low to medium effect sizes. We therefore suggest to regard the detailed discussion of our findings below as the description of trends found in a survey in the general population. These trends may develop more pronounced correlational patterns if they will be applied to clinical populations, as the constructs they target - especially the degree of emotional awareness and the preferred emotion regulation strategy - are of high importance for the investigation and understanding of the psychological processes underlying psychiatric disturbances.

Emotional awareness was negatively related with reappraisal and positively with suppression, the relational pattern between emotion regulation strategy and negative affectivity were congruent between subjects with implicit (level 1 to 2) and explicit (level 3 to 5) emotional awareness, but became especially in women more distinct by dividing the sample into the two LEA-theory derived groups. The third of the sample that demonstrates implicit emotional awareness (n = 533; 28,6%), e.g. reacting to affective arousal with bodily sensations, action tendencies or vague impressions of being under positive or negative tension has a higher correlation between suppression and depression as the majority of the sample who is able to experience and express distinct feelings (LEAS level 3 to 5, n = 1325; 71,31%), whereas in the group with explicit emotional awareness there is a stronger negative correlation between reappraisal and depression. This indicates that the use of adaptive emotion regulation strategies may be fostered by the ability to experience feelings consciously.

In the subsample with subjects capable of explicit emotional awareness (LEAS levels 3 to 5), subjects who reported the use of reappraisal scored lower in negative affect, indicating a relational trend between the ability to be consciously aware of one's emotions and the use of an adaptive emotion regulation strategy (Tab. 3.1). These findings are independent of age and sex, although the pattern of correlations is significantly stronger in women than in men.

In contrast, low emotional awareness may strengthen the relationship between maladaptive emotion regulation strategies and negative affectivity. Independently from age we found low, but highly significant correlations (p<.001) between negative affect and suppression in the subsample with implicit emotional awareness. Epecially women with implicit emotional awareness describe suppression as their main emotion regulation strategy and score high in depression and anxiety.

A path-analysis, combined with a multi-group model comparing subjects with implicit and explicit emotional awareness, indicated that living without a partnership - often identified as a risk factor for mental illness [43], [44] - is related to depression in subjects with low emotional awareness. We used the path-analysis in order to be able to include the possible influence of the demographic variables - which are of high importance in such a large samplein one statistical model and speculate that explicit emotional awareness may moderate the use of adaptive emotion regulation strategies that help people to cope with the hardships of being alone.

In all statistical analyses the above described relations between emotion regulation strategy and negative affect were stronger for depression. Additionally, no direct correlations were observed between the LEAS and depression for the sample as a whole as well as for the subsamples with implicit and explicit emotional awareness. In contrast, we found a positive correlation between the LEAS and self-reported anxiety for the whole sample and for men in the subsample with explicit emotional awareness. This finding - the LEAS scores correlate with self-reported anxiety but not with self-reported depression - replicates findings with the LEAS in a large clinical sample [26] and therefore underlines the validity of the abbreviated LEAS version that we have used in the survey.

The validity of our data is strongly related to the question of whether the short version of the LEAS, which we developed in order to make the collection and the scoring of the free-text answers to the LEAS vignettes feasible for a population-based study with nearly 2,000 participants, is sufficiently correlated with the original 20 item LEAS.

There are three indicators for the validity of the newly developed 4-item version of the LEAS: 1) It has been empirically derived from the data of a large US community sample with a cultural identity comparable to the German population, therefore the socio-cultural reaction patterns that could influence the free-text answers to the emotion provoking social interactions in the LEAS vignettes will be comparable; for culturally informed differences in the reaction to the LEAS vignettes see [45]; 2) in the pilot studies the 4-item LEAS version A demonstrated similar correlational patterns with other psychological constructs as the 20-items or 10-items-LEAS, and 3) the 4-items LEAS replicated in the German general population similar relationships with sociodemographic variables and self-report measures for negative affect as previously reported in the literature [26], [28].

Concluding, we want to note that our study on a representative sample of the German general population gives first empirical support for the assumption that the use of consciously available emotion regulation strategies could be moderated by the level of emotional awareness. The use of adaptive strategies like reappraisal may be linked to the ability to process affective arousal into consciously experienced emotions - the awareness of distinct feeling states could be a pre-condition for thinking about the situation that caused the negative feelings and for taking new perspectives that may change one's mood. The coincidence between our finding that a third of the general German population is characterized by implicit levels of emotional awareness and the data from representative health surveys [46], [47] that identify up to a third of the German population as suffering from psychiatric conditions gives way to the assumption that implicit emotional awareness may be a risk factor for ill mental health. Although of low effect size, the different patterns of significant correlations found in the groups with implict and explict emotional awareness encourages the further exploration of a disease model that hypotheses that impaired emotional awareness obstructs the identification of distinct feelings that may be indispensable for the activation of adaptive emotion regulation strategies acting as protective factors for good mental health.

Further testing of this model in experimental and clinical settings will be required; this studies will also overcome a main shortcoming of our study by applying more sophisticated measures for negative affect and emotion regulation strategies than has been possible in a large representative sample. Other limitations of the presented study lie in its cross-sectional design, which does not allow to draw causal interferences, and in the adoption of a performance measure to a large community study that forces to use an abbreviated version of the measure instead of the more differentiated information that could be drawn from the original 20-item version of the LEAS.

## Supporting Information

Appendix S1 4 items short version of the Levels of Emotional Awareness Scale.
(DOC)
