# Supplementary material for: How Is Emotional Awareness Related to Emotion Regulation Strategies and Self-Reported Negative Affect in the General Population?
Source: PLoS One. 2014 Mar 17;9(3):e91846. doi: 10.1371/journal.pone.0091846 (PMC3956759; doi:10.1371/journal.pone.0091846)
Supplement: Appendix S1 — 4 items short version of the Levels of Emotional Awareness Scale. (DOC) [file pone.0091846.s001.doc]

**Appendix:**

Appendix: 4 items short version of the Levels of Emotional Awareness Scale

Instruction: Please describe what you would feel in the following situations. The only requirement is that you use the word “feel” in your answer. You may make your answer as brief or as long as necessary to express how you would feel. In each situation there is another person mentioned. Please indicate how you think what the other person would feel as well.

[1] You have been working hard on a project for several months. Several days after submitting it, your boss stops by to tell you that your work was excellent. How would you feel? How would your boss feel?

[2] Your boss tells you that your work has been unacceptable and needs to be improved, How would you feel? How would your boss feel?

[3] You and your friend agree to invest money together to begin a new business venture. Several days later you call the friend back only to learn that she/he changed her/his mind. How would you feel? How would your friend feel?

[4] You fall in love with someone who is both attractive and intelligent. Although this person is not well off financially, this doesn’t matter to you – your income is adequate. When you begin to discuss marriage, you learn that she/he is actually from an extremely wealthy family. She/he did not want that known for fear that people would only be interested in him/ her for his/her money. How would you feel? How would she/he feel?
